# Poisoning by Arsenous Acid Ultimately Detected by Examination of the Liver

**Published:** 1843-09

**Authors:** 


*Poisoning by Arsenious Acid ultimately detected by Examination of the Liver.—To the Editor of the Lancet.*—Sir,—Having been for some years past an attentive reader of your valuable Journal, and having received much pleasure and instruction from its perusal, I consider myself bound to communicate to it, in return for my enjoyment, what little information it may be in my power to contribute.

The case which accompanies this note was to me highly interesting, and presented several facts that were worthy of reflection. Perhaps the most important is the discovery of arsenic in the liver in great abundance, after numerous experiments upon the stomach and its contents had failed to detect it; thus illustrating the important medico-legal fact, first promulgated by Orfila, that this poison *often disappears from the stomach by absorption, and becomes separated from the blood of the portal system by the liver*, and that it might be detected there, even after all evidence of its presence has elsewhere vanished. I regret that no opportunity occurred of examining the urine, since the bladder was empty; but evidence existed of inflammation of the mucous membrane of the pelvis of the kidney and ureters. Could that have resulted from the action of this poison? Believe me to be, Sir, your's most obediently, William Bird Herapath.

Pupil of the Bristol Medical School.

Old Park, May 10, 1843.

*Post-mortem appearances. The stomach and its contents; various white matters found therein; great inflammatory action on its mucous surface. Effusion of coagula. The ovaries encysted, and the Fallopian tubes closed and dilated into serous sacs. Local inflammation of the peritoneum. Fluidity of the blood in the heart's cavities. Congestion of the lungs. Chemical experiments on the fluid contents of the stomach, on a portion of that viscus, and on the various white matters and specks found in it, and upon the blood of the right side of the heart but little arsenic found so far; arsenic found in the liver, in much greater quantity.*

June 14th, 1842.—Jane Burns, ætat. 31, having died suddenly at her lodgings, and under suspicious circumstances, Mr. Grindon, the coroner, ordered a post-mortem examination of the body to be made. Mr. Ormerod, having been the medical man in attendance upon the deceased, was instructed to perform it, and having called upon me to conduct the chemical examination of the case, I requsted to be present at the opening of the body, which we found in an upper story of a lodging-house kept by a Mrs. Brown, living in Newfoundland-street, St. Paul's. Its exterior presented no marks of violence; it was much discoloured in the dependant portions by the gravitation of blood. It was fat and plump, and in very good condition, and presented the appearance of a party dying in the prime of life. Death could only have recently taken place, since the limbs were still rigid. The body was opened in the usual way, and the contents of the thorax and abdomen were exposed.

*The lungs* were full and prominent; they did not collapse upon allowing the pressure of the air to exert its influence; not emphysematous; no tubercles seen, if we except a small cretaceous one in the base of the lower lobe on the left, the size of a pea. The lungs were engorged with blood almost as if asphyxia had caused death; upon cutting into them it flowed out in quantities. The black pulmonary substance was deposited in its usual situation; two or three small black bodies, as large as sparrow-shot, were seen in the interlobular fissures, probably enlarged lympatic glands blackened by this substance.

*The pericardium* contained only a teaspoonful of serum.

*The heart* large and flabby; right auricle and ventricle distended with black fluid blood; this was collected for experiment, about six ounces were obtained; the left cavities also contained blood, but not to the same extent, this was also preserved with the other; no coagula on either side. Valves: lining membranes all healthy.

*The pleuræ* contained several ounces of serum, but this was, through an oversight, not saved for examination.

*The abdominal cavity and its viscera.*—The peritoneal surface of the small intestines had a beautiful pink tinge, similar to the hue of a maiden's blush rose; several vessels very distinctly marked. Omentum fat, but healthy; upon pulling up the small intestines (a few coils of the ileum) from the cavity of the pelvis, several bands of organised false membranes were found connecting them with the uterus and with each other; these bands were highly vascular and organised; the peritoneal disease was local, extending only to about the five or six inferior convolutions of the ileum, and were consequently found on the right side. The cœcum was *not* affected in this way, nor its appendix. Other intestines healthy in external appearance.

*The stomach* was next removed, having previously placed ligatures upon its œsophageal and pyloric orifices, removing about an inch of the œsophagus and duodenum at the same time. Upon opening this organ it was found to contain about half a pint of thick grumous fluid, yellowish-red in colour, some half-digested portions of food in it, too much altered to be recognised. These contents were preserved in clean bottles and sealed. The mucous surface of the stomach presented several appearances worthy of notice; its greater cul-de-sac, and a portion of the greater curvature, had a dark livid-red colour; upon closer examination, a clot of blood was seen to have been effused upon its surface and also beneath the mucous membrane. The rugæ were very distinctly marked, and many of them deeply injected and highly inflamed; its pyloric portion was scarcely affected, but the mucous follicles were enlarged, and numerous *white specks* were sprinkled over its surface. Upon raising the coagulum, the mucous membrane was highly inflamed and much softened, being easily torn by the nail. Throughout every part of the stomach these white specks were seen, but they were more numerous in the foveolæ or pits between the rugæ. Some *patches* of white matter were observed upon the coagulum before spoken of, some were also scattered upon the suface of the lining membrane of this organ; but a *peculiar white, shining,* (apparently) crystalline body was seen everywhere diffused. (There was another white body present, but this was *not seen* at this time.)

The stomach was now carefully placed in a new bladder, and reserved for re-examination at more leisure.

The duodenum was removed and examined, but contained only a little fluid, and its appearances were not much worthy of remark. The jejunum contained some fluid and gas; about five ounces were preserved like those of the stomach. One or two red spots upon its mucous membrane, but considered unimportant. The ileum was not examined through its whole length; those portions which were opened presented little worthy of remark, even the membrane of that part whose peritoneal coating was so inflamed, was perfectly healthy. The large intestines not minutely examined. The liver was gorged with blood, but healthy in structure; about half a pound was preserved for subsequent examination. Soon after this I found upon my finger a distoma hepatica, or fluke, it was still alive, and had the usual greyish colour of those found in the liver of the sheep; no others could be seen.

*The pelvic viscera* were next examined.

The finger inserted into the vagina reached the neck of the uterus without difficulty; it was large and well marked; the os tincæ large and elongated; although not pregnant, she might have had a child; she could not have had *more*, as the skin of the abdomen presented none of those seams so well known as the results of pregnancy.

The uterus was of the normal size and unimpregnated; upon its exterior the remains of the false membranes were found which had connected it with the convolutions of the ileum. The Fallopian tubes and right ovary were rather remarkable, and appeared as if they contained an extra-uterine conception. The whole contents of the pelvis were, therefore, taken out with care for closer examination. The bladder was empty. This was to be regretted, as valuable indications might have been obtained of the presence of arsenic in the urine. The kidneys were congested with blood, and the calyces contained some muco-purulent fluid, and apparently the mucous membrane was inflamed, as it was highly vascular. Was this the result of gonorrhœa or of arsenic? no other evidence of the former disease existed. I am inclined to the latter opinion, but cannot prove its truth.

To return to the genital organs. The uterus upon being opened was healthy and empty; the mouth admitted the handle of a small scalpel easily. The Fallopian tubes were both encysted, the fimbriated extremities being adherent to the recto-uterine fold of peritoneum on the left, but to the enlarged ovary on the right. Their ostea-uterina were obliterated, the adhesions were probably old, as they were organized. The right tube being opened, about two or three drachms of yellow serous fluid escaped; very little fluid flowed from the left, scarcely a drachm, but no fœtus in either. The left ovary contained several cavities; one seemed to communicate with the peritoneal cavity, as an opening was found having rounded edges and not appearing as if cut, this cavity would have lodged a pea; the internal surface was slightly yellow; all the sacs were empty and communicated; three or four were found.

The right ovary was at first, from its feel and appearance, supposed to contain an extra-uterine conception, and it was opened rather reluctantly; it evidently contained a fluid and a solid; this was rounded and not well defined. Upon making a puncture, about two ounces of reddish serum (sero-sanguinous fluid), escaped, and a solid body with a shaggy surface was seen blocking up the orifice; this was, therefore, enlarged and the body turned out; it was found to be a coagulum of blood (?) homogeneous in texture, but yellowish within. The walls of this cyst were lined by a false membrane, upon the removal of which the serous shining surface of the cyst appeared. The whole ovary was as large as a tennis-ball. The peritoneal coat presented the remains of the false membranes attached to it, thus holding the ileum down firmly, as before stated. The walls of the ovary only one-sixteenth of an inch thick, and *none* of the healthy structure of the ovary remained.

Did the peritoneal inflammation result from the rupture of a similar cyst in the left ovary and the escape of the fluid into the peritoneal cavity? I am inclined to think that it did. It is clear that she could not have been pregnant, and doubtful if she ever would have been again, had she lived.

The woman is a native of Ireland; her maiden name was Maggs. She married a man named Wright, who died; she then married a sergeant (now in India), but she formed a disreputable connection with a man living at Cathay, and was in the habit of meeting him every Saturday night, at a public house, and he then usually accompanied her home; but on the 11th instant they quarrelled, and he did not go home with her. On Sunday morning she was found ill, having previously written some letters with pen and ink borrowed from her landlady, together with a prayer-book; some time after this a little girl was sent up stairs to fetch these things back and found the door locked on the inside. By-and-by the landlady heard a noise up stairs, and found the deceased very ill, and groaning terribly. She fetched her neighbour, and then the woman asked for water, and soon afterwards was sick, and "vomited a large quantity of white stringy fluid," which they foolishly threw away. To a medical man who was sent for she complained much of her heart, and appeared in great agony, rolling and tossing in her bed, but he could gain very little information from her. She either would not or could not answer his questions. Before any medicine could be prepared she died. In her room he found in her reticule a small paper packet (with "arsenic, poison," written on the outside), containing a white powder.

She had only been in the house a week, and upon taking the lodgings had stated that her husband lived in Bath, but came home on Saturday nights, and would then pay the rent. She added that she believed herself to be *enciente*, but the post-mortem examination clearly proved that this suspicion was unfounded. It was evident that she could not have been recently delivered. It is doubtful whether she *ever* had a child, although the uterine orifice was not in its virgin state. The disease in the ovaries and Fallopian tubes would prevent her from becoming pregnant. Although it is not very probable that this was a congenital malformation, yet it must have existed for some time, perhaps for several years. The peritoneal disease must have been old too, since the membranes were organised, and no recent lymph or fluid was effused.

The congested state of the lungs and the engorgement of the right side of the heart, would seem to point to asphyxia as the immediate *precursor*, if not the *cause* of death.

But the gastric symptoms must have been acute with such extensive disease; the inflammation was intense, blood and coagulable lymph having been effused, together with softening of the mucous membrane, in patches, all being evidences of acute gastritis, and appearing to point to a corrosive poison as the proximate cause. The abundant presence of white particles, &c., in the stomach, seemed to support this conclusion.

Under these circumstances I commenced the chemical examination with alacrity, expecting to find it in abundance, and in every portion used.

I first assured myself, by reducing the metal, that the substance *contained in the paper* found in her reticule was arsenious acid; again oxidising it, and obtaining the green arsenite of copper, the yellow sulphuret of arsenic.

A. I took a portion of the fluid contents of the stomach.

About four ounces were evaporated to dryness (during the boiling a strong smell of toasted cheese was perceptible); it was next mixed with nitre, and deflagrated in a pure silver dish. When all the animal matter was destroyed, and the dish and contents allowed to cool, it was treated with water until dissolved, then filtered. To the clear solution acetic acid was added in small portions, until a slightly acid reaction appeared evident to litmus paper, carbonic acid being evolved; a current of sulphuretted hydrogen gas was then passed through this. A brownish-white was thus obtained. The fluid was now boiled to expel the excess of hydrosulphuric acid and to assist the coagulation of the precipitate. It was now set aside, in order that the precipitate should fall. Next day the supernatant fluid was removed by the pipette, and the precipitate dried. It was then treated with a little water, and got into a smaller compass, and again allowed time to subside. This water was again removed by the pipette, and I again dried the precipitate. Having now well dried some charcoal and carbonate of soda, small portions of these substances were mixed intimately with half of the precipitate, and introduced into Berzelius's reducing tube by means of a small funnel, and then, upon cautiously applying heat, so as to expel a small portion of water (which always remains notwithstanding all your care), and having removed this by means of a small coil of bibulous paper introduced into the tube for that purpose, the bulb of the tube was heated to bright redness; a little sulphur rose or sublimed. Beneath this ring of sulphur the merest trace in the world of a *dark ring* was perceptible, but this was not sufficient to be able to say that it was arsenic, although a *very suspicious* circumstance. (I preserved this.) The second portion, treated in the same way, gave similar results. Upon endeavoring to pass this portion through the usual series of tests for arsenic in solution, no results were obtained.

B. In the intervals occurring in the progress of this experiment I took about two or three inches square of the *pyloric extremity* of the stomach (here, be it observed, the white specks were most numerous) and dried it in an oven, having previously washed its surface, to obtain the white particles. The washings were evaporated to dryness, and treated precisely in the same way as the last, but not the slightest trace of arsenic was perceived.

C. The solid portion of the stomach from B was treated exactly in the same way. The residue was subjected to the same process as in A: a slight smoky tint only obtained by reduction, but *far* from being conclusive. I preserved this as a specimen.

D. During the spare time, whilst these experiments were on hand, I took four or five ounces of the blood, evaporated to dryness, constantly stirring it, and treated this by the same process. Had I been able to forsee the *results* of these experiments I should not have tried this, as these were still *in umbrâ*. I commenced this merely as an experiment from curiosity, as all the previous experiments could not be finished until the next morning. This was treated precisely as before, and in the morning it was reduced, but nothing rose, a ring of sulphur only subliming,

E. Having so signally failed in all these trials I began to feel annoyed; for being morally certain that arsenic had caused death, why was it not found? The process had proved successful over and over again in my hands, and my father has been eminently successful with it. Then why was it fallacious here?

I now searched for other poisons, and as the mineral acids were likely bodies, these formed the indices for experiments, but none were present.

Then it occurred to me to examine more closely the substances still adhering to the surface of the stomach. Had all these white specks, &c., been arsenic, not the least difficulty could have happened with regard to them.

First. Multitudes of small, white, shining, crystalline globules, were present, but of a very *small* specific gravity, and having a silky lustre. Placed on a Stanhope lens they were very transparent, and not unlike granules of starch; but tincture of iodine gave no indication of that substance. They were insoluble in acetic acid; ammonia, boiling, partially dissolved them, as did potash. Were they fatty grains? I think so. They were everywhere seen upon the stomach, more in the pits than on the rugæ, *floating on the fluid.*

Second. A more opake white substance, found near the pyloric end, more like arsenious acid, but when placed on the Stanhope lens they proved to be soft and plastic, were not gritty between the nails; when placed in a small tube and heated they intumesced, charred, and smelt strongly of toasted cheese. A crystalline white body sublimating, this was not soluble in water, and the water, when tested for arsenious acid, gave a *blue* precipitate with the ammoniacal sulphate of copper; they were not arsenic, neither did they contain any.

Third: A third white substance; white patches: these were apparently fibrinous. Took a portion of the coagulum with these patches upon it, boiled in strong nitric acid, again and again, until no red fumes of nitrous acid evolved. then evaporated to dryness, A fatty body left; upon adding ammonia, an orange, soapy compound; this was then treated with acetic acid and sulphuretted hydrogen, as before the precipitate attempted to be reduced, but nothing sublimed.

Fourth. I scraped a portion of the mucous membrane with a spatula; the mixed body, &c., were acted on by nitric acid, as in the last experiment, and neutralised by ammonia. Upon dissolving by the aid of heat, a yellowish soap, as before; upon testing this solution by the copper, silver, and sulphuretted hydrogen tests, something like the precipitates were obtained. However, as these could not be depended upon, as the animal soap was present, this was deflagrated with nitre, as in A (after previous evaporation to dryness), and being treated as before, nothing rose.

Fifth. I was now obliged to attend the inquest. Having only suspicions of the presence of arsenic, I gave my evidence in a guarded manner, and having detailed these results, requested an extension of time; but the coroner and jury thought, notwithstanding my having stated my belief that it was present, that nothing would be gained by the delay, especially as the evidence all tended to prove that she had committed suicide. They therefore returned a verdict of died "from inflammation of the stomach." Upon my return home I again set to work, and having poured a fine stream of water upon the stomach so as gently to wash off these light particles, so embarrassing, I took it into the sunlight, and carefully went over the whole mucous membrane (of the stomach) with a lens, inch by inch, By this means several small grains of a white body were obtained, very different from those previously found, appearing as opake white specks, very sparingly scattered over its surface and closely adherent to it. Their margins were more defined than any yet seen, its edges being sharp. Being observed amongst the patches of different white substances previously found, they stood somewhat boldly forward, inviting attention. Having, after a good deal of trouble, picked out with the point of a pin and the help of the lens about twenty or thirty of them, placing them one by one, as I did so, in a watch-glass with a little water, some fell down, as heavy bodies would do, others swam and floated away. At length, having sufficient of the heavy ones, I decanted the fluid and lighter particles, washing those remaining in the watch-glass again and again with a little cold water. They were then dried and mixed with carbonate of soda and charcoal, as before, and having introduced the mixture into a reducing tube, upon heating it in a short time the metallac arsenic arose in its usual splendour. This I preserved. I again went over the stomaeh, and obtained a second portion of these particles. Some were preserved as specimens, others were reduced and then subjected to the usual tests, as additional evedence of their nature.

F. Not being very well satisfied at having missed this body in the fluid contents of the stomach, I now took the remainder of the contents of the stomach and intestines, about two-thirds of the original quantities found; they were treated as in A. Upon subliming this, I obtained a *slight* indication of metallic arsenic, a ring of fly-powder appearing; the metallic ring *was not* perceptible; it would do as a corroborative evidence only.

G. Took half a pound of the liver; having cut it into small portions and introduced them into a tubulated retort, they were boiled with concentrated nitric acid; violent effervescence took place from the escape of carbonic acid, nitrogen and nitrous acid gases, more and more acid being added from time to time; the fibrinous masses first disappeared; being converted into a fatty substance, this gradually disappeared also, leaving a deep orange-coloured solution; this was evaporated to dryness, and the operation again recommenced as before. When the decomposition had been pushed as far as possible without danger of the explosion which so often occurs towards the end of this process, it was stopped by slow evaporation to dryness again; the residue was dark chocolate-coloured, and weighed about two drachms.

H. This residue was now boiled in a solution of carbonate of potass; effervescence took place, an orange-yellow solution being formed, and the same dark-chocolate residue remaining. This was proved to be sesquioxide of iron with animal matter.

I. The solution was now separated by filtration, and slightly acidulated with acetic acid and a current of sulphuretted hydrogen passed through it: then boiled to expel the excess of this gas and allowed to stand for a time in order that the precipitate should subside; decanted the fluid and washed and dried the precipitate; part of this was now introduced into the reducing tube, as before, when upon heating it an *abundant ring* of metallic arsenic sublimated. This was preserved as a specimen, another experiment gave similar results, and farther, for by oxidising the ring of metal and dissolving in water and applying the tests for arsenious acid in solution, the characteristic precipitates were produced.

K. The residue from H, upon being farther examined, was found to contain no arsenic, but consisted of phosphate of lime, sesquioxide of iron, animal matter, and a portion of silica.

This operation, namely, the destruction of the animal matters of the liver by means of nitric acid, is a long and tedious process; yet it is an interesting one, and, by its results, fully repays one for the trouble, for in this case we see what minute portions of the poison remained in the organs where it is usually sought for. The *fluid* contents contained the merest possible trace, certainly not near enough to enable one to speak positively of its presence, although it is possible to prove the existence of one-thousandth part of a grain of this substance, and very probably even a much less quantity. It is true that by *great care and trouble* several little granules of the poison were obtained *from the coats* of that viscus, but the operation takes too much time to have allowed me to use this process before the inquest. Again, the *best* plan would have been, undoubtedly, to have destroyed the whole stomach by the same process; but the case appeared so clear, and such masses of evidence seemed already present, that this caution was apparently unnecessary.

The discovery of arsenic in the liver, when such small traces exist in the stomach and its contents, appears to be important; highly so, indeed; in fact, giving a means of finding this substance when all other processes have failed; and in *no case* of poisoning ought this viscus to be slighted at the *post-mortem* examination, the same care being used in removing it as in taking out the stomach and other viscera, for in the liver *the whole evidence* might exist.

Now to account for the sparing evidence of poison in the *fluid* contents. Pereira, in his "Materia Medica," vol. i., page 376, says, "Water, boiling, dissolves one twenty-fourth part of its weight of arsenious acid; on cooling it retains one-fortieth part of its weight; but by *cold digestion* it only retains one-thousandth or one five-hundredth of its weight. This is stated upon the authority of Mr. Taylor, in 'Guy's Hospital Reports,' vol. ii., p. 83." Could this have been the cause? I believe that it was. There is no evidence as to *how* the poison was taken, but it might have been swallowed in cold or tepid fluid. It was certainly suspended in fluid, since some granules of it still adhered to the stomach.

